# Ex-vivo expansion of nonhuman primate CD34^+^ cells by stem cell factor Sall4B

**DOI:** 10.1186/s13287-016-0413-1

**Published:** 2016-10-20

**Authors:** Bin Shen, Yu Zhang, Wei Dai, Yupo Ma, Yongping Jiang

**Affiliations:** 1 Biopharmaceutical R&D Center, Chinese Academy of Medical Sciences & Peking Union Medical College, 277 Qingqiu Street, Suzhou, 215126 China; 2 Biopharmagen Corp, Suzhou, 215126 China; 3 Environmental Medicine, NYU Langone Medical Center, Tuxedo, NY 10987 USA; 4 Department of Pathology, BST-9C, The State University of New York at Stony Brook, Stony Brook, NY 11794 USA

**Keywords:** Nonhuman primate CD34^+^ cell, Sall4B, Ex-vivo expansion, NOD/SCID mice

## Abstract

**Background:**

Hematopoietic CD34^+^ stem cells are widely used in the clinical therapy of complicated blood diseases. Stem cell factor Sall4B is a zinc finger transcription factor that plays a vital role in hematopoietic stem cell expansion. The purpose of our current study is to further evaluate how Sall4B might affect the expansion of CD34^+^ cells derived from nonhuman primates.

**Methods:**

Sall4B was overexpressed in nonhuman primate bone marrow-derived CD34^+^ cells via a lentiviral transduction system. The granulocyte–erythrocyte–macrophage–megakaryocyte colony-forming unit (CFU) assay evaluated the differentiation potential of primate CD34^+^ cells that were expanded with Sall4B. Furthermore, an in-vivo murine system was employed to evaluate the hematopoietic potential of primate Sall4B-expanded CD34^+^ cells.

**Results:**

Overexpression of Sall4B promoted ex-vivo nonhuman primate CD34^+^ cell expansion by 9.21 ± 1.94-fold on day 9, whereas lentiviral transduction without Sall4B expanded cells by only 2.95 ± 0.77-fold. Sall4B maintained a significant percentage of CD34^+^ cells as well. The CFU assay showed that the Sall4B-expanded CD34^+^ cells still possessed multilineage differentiation potential. A study using nonobese diabetic/severe combined immunodeficiency (NOD/SCID) mice in vivo revealed that Sall4B led to an increase in the number of repopulating cells and the 9-day-old Sall4B-transduced CD34^+^ cells still possess self-renewal and multilineage differentiation capacity in vivo, which are similar stemness characteristics to those in freshly isolated primate bone marrow-derived CD34^+^ cells.

**Conclusions:**

We investigated the expansion of nonhuman primate bone marrow-derived CD34^+^ cells using the Sall4B lentiviral overexpression approach; our findings provide a new perspective on mechanisms of rapid stem cell proliferation. The utilization of Sall4B to expand CD34^+^ cells on a large scale through use of suitable model systems would prove helpful towards preclinical trials of autologous transplantation.

## Background

Hematopoietic stem cells (HSCs) are rare stem cells that have two defining features: self-renewal and multilineage differentiation. They have the ability to differentiate into specialized blood cells, including lymphocytes, red blood cells, and platelets [1]. HSC transplantation can be an important life-saving strategy in the clinical treatment of a broad spectrum of disorders, such as lymphoma, leukemia, and some other immune and genetic diseases [2–4]. However, the full therapeutic potential of HSCs has not been achieved. The HSC niche, described as the microenvironment, contains stem cells, stem cell progeny, osteoblasts, stromal cells, adipocytes, cytokines, and chemokines. This niche controls the balance between HSC self-renewal and differentiation [5]. However, understanding how niches modulate self-renewal and directional differentiation remains a challenge for scientists worldwide [6].

It is essential to determine how transcription factors affect HSC expansion, and to establish an efficient method for enhancing the intrinsic self-renewing properties of HSCs ex vivo. The homeobox B4 (*HoxB4*) gene belongs to the homeobox gene family and promotes self-renewal and expansion of HSCs ex vivo [7–9]. The manipulation of signaling pathways for genes such as Notch and Wnt [10, 11] has also shown some effects on ex-vivo HSC expansion. Sall4 is a zinc-finger transcription factor and a member of the Sall gene family, which was originally cloned based on sequence homology to Drosophila *spalt* (*sal*) [12–14]. In Drosophila, *sal* is a homeotic gene that is essential for the development of posterior head and anterior tail segments [15]. Recently, we demonstrated that lentiviral expression of Sall4 in human bone marrow (BM) hematopoietic stem/progenitor cells (HSPCs) was able to dramatically expand and enhance their ability for long-term engraftment in nonobese diabetic/severe combined immunodeficiency (NOD/SCID) mice [1, 16, 17]. During normal hematopoiesis, Sall4B plays an important role in HSPCs by promoting self-renewal and inhibiting differentiation [18]. *Bmi-1* is a member of Polycomb Repressive Complex 1 that has been shown to be a critical regulator of hematopoiesis and leukopoiesis [19–21]. A previous study showed that the oncogene *Bmi-1* is a direct target gene of Sall4B, where Sall4B expression strongly correlates with *Bmi-1* in primary acute myeloid leukemia (AML) and high levels of H3–K4 trimethylation and H3–K79 dimethylation were observed in the Sall4B binding region of the *Bmi-1* promoter [22]. Other researchers claimed that Sall4B may act as either an activator or a repressor of *Bmi-1* in a dose-dependent fashion in hematopoiesis. In the presence of extremely low Sall4B expression levels, HSPCs would show loss of self-renewal. However, in the presence of very high Sall4B expression levels, *Bmi-1* might be suppressed, and HSCs would lose their ability for self-renewal and multilineage differentiation. HSPCs could maintain self-renewal, multipotency, and differentiation only when Sall4B expression was balanced appropriately [23].

The nonhuman primate is an important animal model that can be applied to preclinical studies of stem cell transplantation. Here, we demonstrated that Sall4B overexpression could significantly enhance expansion of nonhuman primate BM-derived CD34^+^ cells ex vivo, and also in vivo in NOD/SCID mice. Furthermore, Sall4B overexpression could maintain multilineage differentiation capability and increase repopulating cell number as demonstrated by ex-vivo granulocyte–erythrocyte–macrophage–megakaryocyte colony-forming unit (CFU-GEMM) assay, and demonstrated in vivo in murine models. Our findings would be vital for preclinical studies of nonhuman primate autologous CD34^+^ cell transplantation that use the Sall4B overexpression approach on a large scale.

## Methods

### Ethics statement

All research involving animals was conducted according to relevant national and international guidelines. Female NOD/SCID mice (6–8 weeks old and 16.2–17.3 g) were obtained from the Experimental Animal Center of Soochow University (Suzhou, China). The experimental protocols were approved by the Institutional Animal Care and Use Committee of Soochow University (IACUC permit number: SYXK(Su) 2014-0078).

The male cynomolgus primate (6 years old and 5.7 kg) whose BM was used was obtained from the Medical Primate Research Center of the Institute of Medical Biology, Chinese Academy of Medical Sciences. The primate was housed and bred according to the guidelines of the Experimental Animals Ethics Committee at the Institute of Medical Biology, Chinese Academy of Medical Sciences. The experimental protocol was also reviewed and approved by the Yunnan Province Experimental Animal Management Association (Permit Number SYXK-YN No. 2014-0017) and the Experimental Animal Ethics Committee of the Institute, which complied with the humane regulations of replacement, refinement, and reduction (3Rs). For BM sampling, nonhuman primate was anesthetized with ketamine/acepromazine at a dosage of 0.1 ml/kg body weight, intramuscularly, prior to handling and BM puncture (in accordance with institutional standard operating procedures).

All surviving animals were also euthanized at the study endpoint under anesthesia using a pentobarbital-based euthanasia solution.

### Animals

NOD/SCID mice were housed in individual stainless steel cages in a SPF facility in Soochow University, with a regulated temperature of 24 ± 2 °C, relative humidity of 50 ± 10 %, and a 12-hour light cycle. Mice were sacrificed by carbon dioxide (CO_2_) inhalation at 8 weeks post transplantation. Peripheral blood (PB) and BM were collected immediately after euthanasia.

The primate from which BM was obtained was housed in an adjoining individual primate cage (130 cm × 53 cm × 80 cm) allowing social interactions, under controlled conditions of humidity, temperature, and light (12-hour light/dark cycle, 7:00 am–7:00 pm). Food and water were available ad libitum. Environmental enrichment consisted of commercial toys. The primate was maintained at approximate free-feeding weight by postsession feedings of a nutritionally balanced diet of high-protein banana-flavored biscuits. In addition, fresh fruit and environmental enrichment were provided daily. The primate was monitored twice per day by the veterinarians. In cases of suffering, the primate was treated with analgetic drugs.

### Lentivirus production and titration

**Fig. 1:**
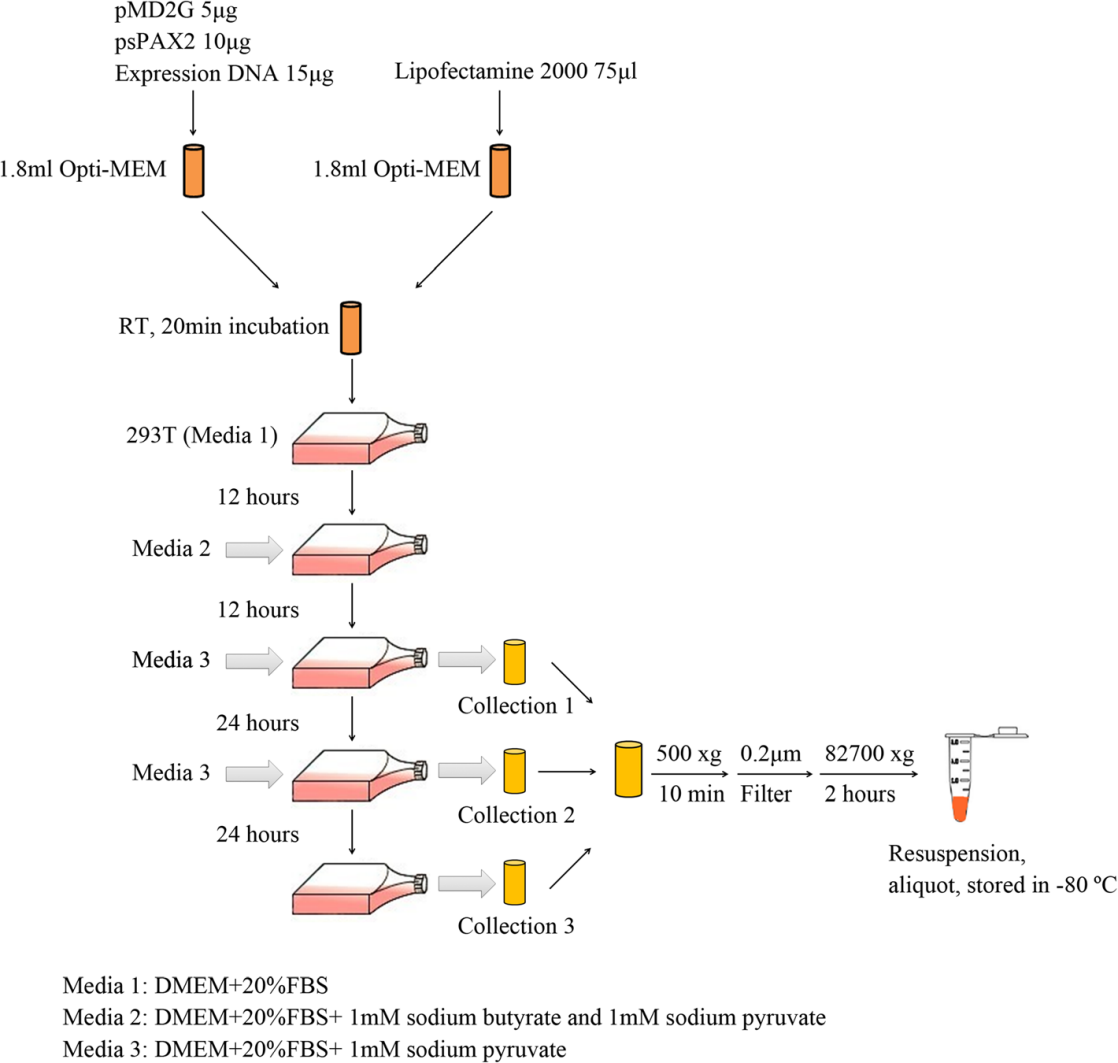
Flow chart of lentivirus titration and production. Optimized lentivirus packaging protocol for production of high-titer lentivirus

An optimized lentivirus packaging protocol was developed to harvest high-titer green fluorescent protein (GFP)-Sall4B lentivirus and GFP-control lentivirus [24]. As shown in Fig. 1, 293 T cells seeded in a T75 flask were used to package lentiviruses. Four high-quality vectors (pMD2.G, pSPAX2, GFP-Sall4B plasmid, and GFP-control plasmid) were obtained through a bacterial expression system. Sodium pyruvate and sodium butyrate that were added to the media after DNA transfection significantly improved the lentivirus yield. Three sample collections were performed after transfection at 12, 36, and 60 hours. Collected samples were filtered through a 0.2 μm filter and centrifuged at 82,700 × *g* for 2 hours. Finally, the lentivirus pellet was suspended with 200 μl IMDM. Lentivirus titer was determined with the Lenti-X™ qRT-PCR Titration Kit (Clontech) according to the manufacturer’s instructions. The lentivirus was aliquoted and stored at –80 °C until use for transduction.

### Nonhuman primate CD34^+^ cell isolation

CD34^+^ cells were enriched from fresh total mononuclear cells (MNCs) of nonhuman primates with the magnetic-activated cell sorting (MACS) immunomagnetic absorption column separation device, coupled with mouse anti-primate CD34 antibody (BD, USA) and anti-mouse IgG MicroBead (Miltenyi Biotec, Germany), according to the manufacturer’s instructions. MNCs were obtained from fresh nonhuman primate BM using density centrifugation with Ficoll-Hypaque Premium (GE Healthcare, USA). The purity of CD34^+^ cells was verified using flow cytometry, with anti-primate CD34 mAb conjugated with phycoerythrin (PE; Immunotec, Canada) and the BD FACSVerse flow cytometer (BD, USA).

### Determination of optimal multiplicity of infection

Freshly isolated BM-derived primate CD34^+^ cells were seeded in 24-well plates at a density of 2 × 10^5^/ml. The cells were cultured in StemSpan SFEM (Invitrogen, USA) containing 5 % FBS and 1 % penicillin/streptomycin. The media were supplemented with 100 ng/ml Flt-3 L (PeproTech, USA), 20 ng/ml thrombopoietin (TPO) (PeproTech, USA), and 100 ng/ml stem cell factor (SCF) (PeproTech, USA). The next day, GFP-Sall4B lentivirus was added with 5 μg/ml polybrene (Millipore, Billerica, USA) to each well at different multiplicities of infection (MOIs) of 0, 5, 20, 80, and 200 (*n* = 3). For controls, GFP-control lentivirus was added to the CD34^+^ cells at a MOI of 20. The cells were transduced overnight for 12 hours and then recovered in fresh culture media. Media were changed every other day for cell growth. On day 9, cells were collected for cell count and flow cytometry in order to determine the accurate number of total nucleated cells and CD34^+^ cells in each group. Furthermore, western blot analysis of Sall4B was performed to determine the Sall4B protein expression level in each group.

### Lentivirus transduction and cell expansion

The isolated BM-derived primate CD34^+^ cells were cultured in 24-well plates overnight in StemSpan SFEM supplemented with the mentioned three cytokines (SCF, TPO, and Flt-3 L). GFP-Sall4B lentivirus and GFP-control lentivirus were added at MOI of 20 on the following day, supplemented with 5 μg/ml polybrene. Twelve hours later, the media were replaced, and were subsequently changed every other day until day 9. Cells were then collected and prepared for cell count, flow cytometry, and study in vivo*.*

### Colony-forming unit assay

MethoCult H4230 methylcellulose media (Stem Cell Technologies, Vancouver, Canada) were thawed overnight at 4 °C in a refrigerator. Unexpanded primate CD34^+^ cells, day 9 GFP-control, and GFP-Sall4B-expanded CD34^+^ cells were prepared at the required final plating concentration of 1 × 10^4^ cells per dish. Duplicate cultures were prepared with 1.1 ml cell suspension in each 35-mm dish. The cells were incubated at 37 °C in 5 % CO_2_ with >95 % humidity for approximately 16 days. The various CFUs, including erythroid burst-forming unit (BFU-E), granulocyte colony-forming unit (CFU-G), granulocyte–monocyte colony-forming unit (CFU-GM), megakaryocyte colony-forming unit (CFU-M), and CFU-GEMM, were analyzed with a bright-field microscope on day 16 after the cells were plated in MethoCult media. A colony with >100 cells was counted as a positive colony.

### NOD/SCID mice transplantation and repopulating assays

NOD/SCID mice received sublethal irradiation of 250 cGy from a ^60^Co source (radioactive intensity: 0.38 Gy/min) 24 hours before cell transplantation. All mice were randomly divided into five groups (*n* = 12 per group). The negative control group was injected with 150 μl saline. The unexpanded group was transplanted with 2 × 10^5^ freshly isolated primate CD34^+^ cells. The other groups were transplanted with day 9 expanded primate CD34^+^ cells from 2 × 10^5^ starting cells (about 6 × 10^5^ CD34^+^ cells per mouse), day 9 GFP-control lentiviral transduced cells from 2 × 10^5^ starting cells (about 6 × 10^5^ CD34^+^ cells per mouse), or day 9 GFP-Sall4B lentiviral transduced cells from 2 × 10^5^ starting cells (about 1.8 × 10^6^ CD34^+^ cells per mouse). Saline or cells were transplanted by tail vein injection. PB samples were collected from the retro-orbital plexus and BM samples were harvested from both femurs/tibias of eight mice for each group in week 8 post transplantation. PB and BM cells were stained with antibodies and analyzed by flow cytometry. Briefly, mouse blood samples were treated with red blood cell lysis buffer to remove red blood cells while preserving the leukocytes. Treated cells were then washed with phosphate-buffered saline (PBS) and incubated with PE-CD45, allophycocyanin (APC)-CD14, or fluorescein isothiocyanate (FITC)-CD20 antibodies (BD Pharmingen™) at room temperature (RT) for 15 min in the dark. Cells were labeled with mouse IgG isotype control monoclonal antibodies. The physical conditions of the remaining four mice of each group were observed for 6 months. The frequency of SCID repopulating cells (SRC) was determined in limiting dilution assays using the method of maximum likelihood with L-CALC™ software (StemCell Technologies, USA) from the proportions in the engrafted recipients (≥0.5 % primate CD45^+^ cell engraftment, *n* = 30) measured in the groups of mice transplanted with the progeny of different numbers of starting cells for groups of unexpanded CD34^+^ cells, 9-day-old GFP-control lentivirus transduced CD34^+^ cells, and 9-day-old GFP-Sall4B transduced CD34^+^ cells.

### Serially transplanted studies

Mouse BM cells were harvested from the femurs of highly engrafted primary recipient mice 8 weeks after transplantation. After removal of red blood cells, total BM cells were transplanted into the secondary sublethally irradiated (2.5 Gy, radioactive intensity: 0.38 Gy/min) NOD/SCID mice (*n* = 8 per group). Eight weeks after transplantation, the percentage of nonhuman primate CD45^+^ cells in PB of the secondary recipient mice was analyzed by flow cytometry.

### Statistical analysis

One-way analysis of variance (ANOVA), followed by Dunnett’s multiple comparison test, was used for comparisons among the various groups. Results were considered statistically significant when *P* < 0.05.

## Results

### Titration of lentivirus packaging

**Fig. 2:**
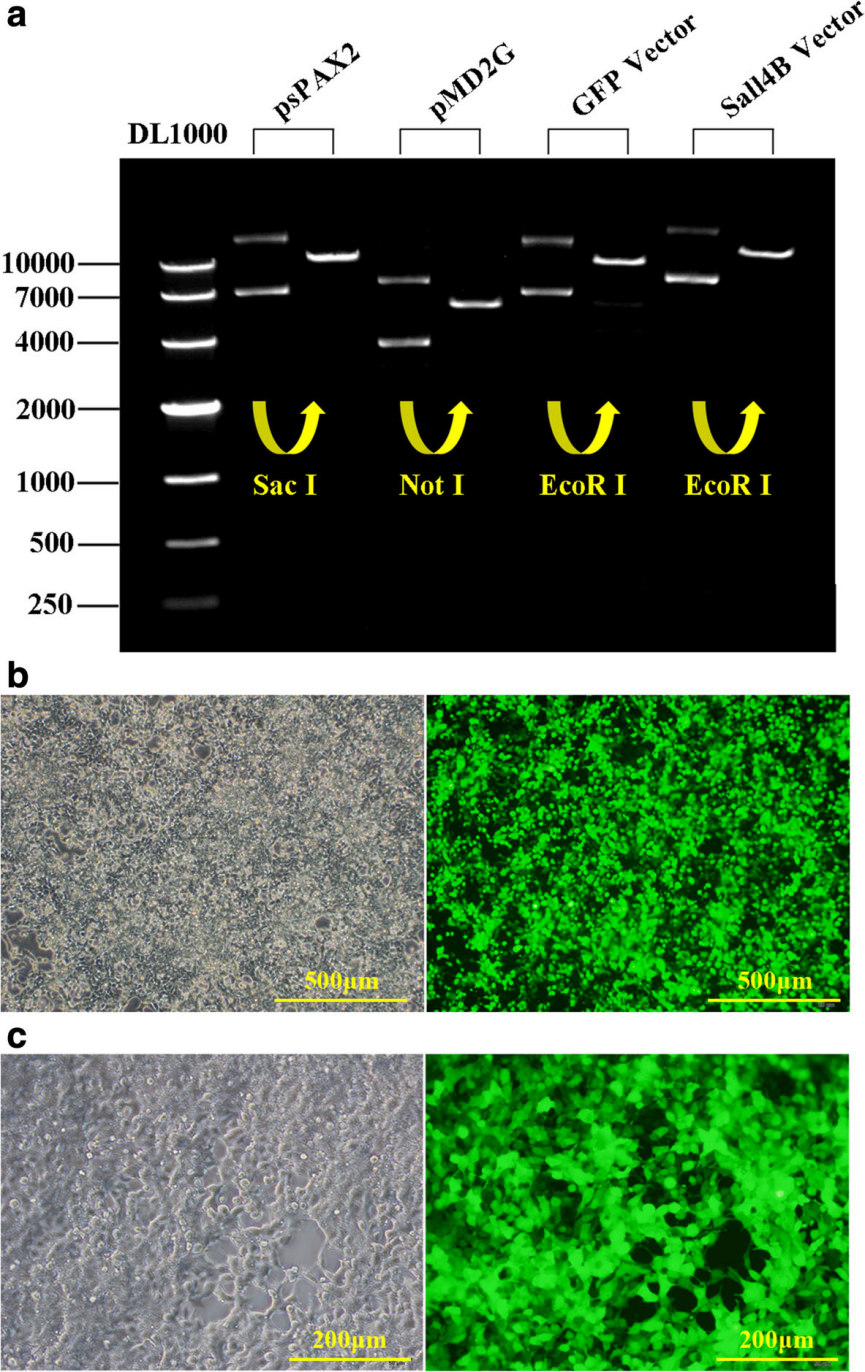
Quality control of vectors and GFP expression in 293 T cells. **a** Four plasmids (psPAX2, pMD2.G, GFP-control expression vector, and GFP-Sall4B expression vector) were amplified by bacteria amplification system and linearized by single digestion. The restriction endonuclease recognition sites were *Sac* I, *Not* I, *EcoR* I, and *EcoR* I. **b** 293 T cells were monitored after 12 hours during the lentivirus packaging process (10 ×). **c** GFP expression in 293 T cells 12 hours after lentivirus transduction (20 ×)

Vectors for packaging were extracted with a plasmid kit (QIAGEN, Germany). Purity of the single digested plasmids was assessed (Fig. 2a). The OD260/280 ratio was in the range of 1.8–1.9, suggesting high-purity DNA extraction for lentivirus packaging. Packed lentiviral particles were collected at 12, 36, and 60 hours, as shown in Fig. 1. The 293 T cells showed abnormal size after 60 hours. The high green fluorescence intensity (Fig. 2b) observed after 48 hours indicated the high transfection efficiency of 293 T cells. Various cell lines were used to test the transduction efficiency of lentivirus, including 293 T cells (Fig. 2c), HeLa cells, and endothelial cells (data not shown). Strong fluorescence intensity indicated the high lentivirus titer, which was essential for CD34^+^ cell transduction. Cell toxicity was avoided by determining the specific MOI and transduction time of different cell types. In general, the transduction time for carcinoma cell lines (293 T and HeLa) was about 8–12 hours with a MOI range of 1–10, which would be optimal to reach high transduction efficiency and low cell toxicity. An average lentivirus titer of 4 × 10^8^–5 × 10^8^ IU/ml could be obtained using the Lenti-X™ qRT-PCR Titration Kit.

### Nonhuman primate BM CD34^+^ cell isolation

**Fig. 3:**
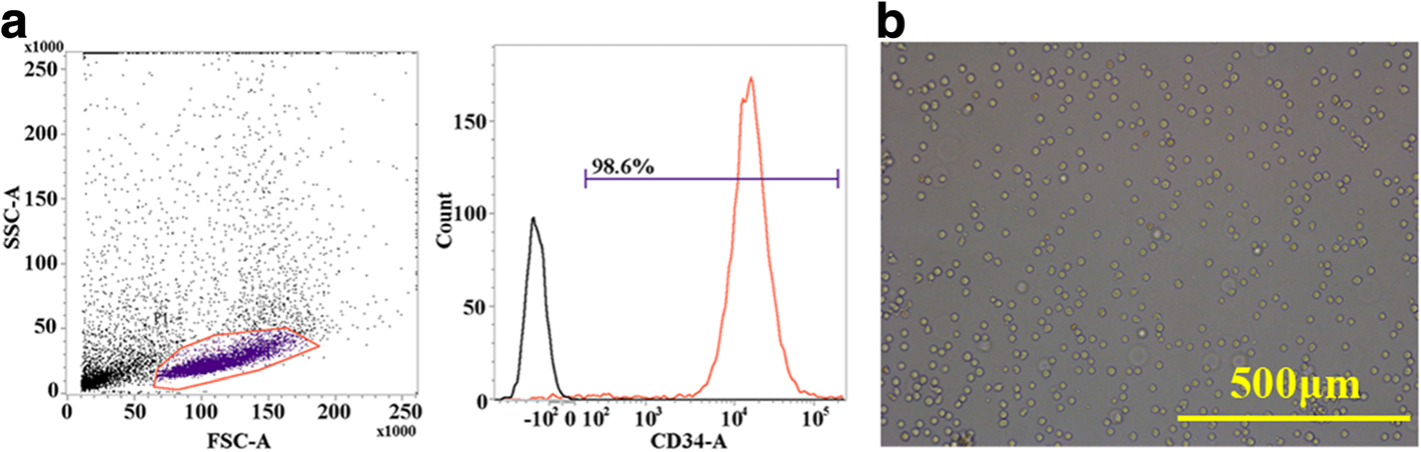
Nonhuman primate BM CD34^+^ cell isolation. **a** Purity of isolated primate CD34^+^ cells. CD34^+^ cells were isolated via magnetic bead-based antibody affinity purification as described in Methods. (*Left*) Scatter-plot representation of purified CD34^+^ cell preparations. Forward scatter (*FSC*) and side scatter (*SSC*) were used to define gate MNC. (*Right*) (*black curve*) Negative control. The signal was detected using a fluorescently-labeled APC and isotype-matched monoclonal mouse IgG control antibody. (*Red curve*) Purified cells incubated with fluorescently-labeled APC mouse anti-nonhuman primate CD34 mAb. **b** Cell morphology of the freshly purified nonhuman primate CD34^+^ cells (20 ×)

Primate BM was obtained by tibial BM aspiration. About 6–8 ml of total BM could be gained in one aspiration (3–4 ml per tibia). After MACS cell isolation, a high percentage of CD34^+^ cells was harvested. The ratio of CD34^+^ cells to whole cells was 98.6 % as determined by flow cytometry (Fig. 3a). The isolated CD34^+^ cells were cultured in 24-well plates at a density of 2 × 10^5^/ml and recovered overnight. The purified cells were homogeneous when observed by microscopy (Fig. 3b).

### Determination of optimal MOI for GFP-Sall4B lentivirus

**Fig. 4:**
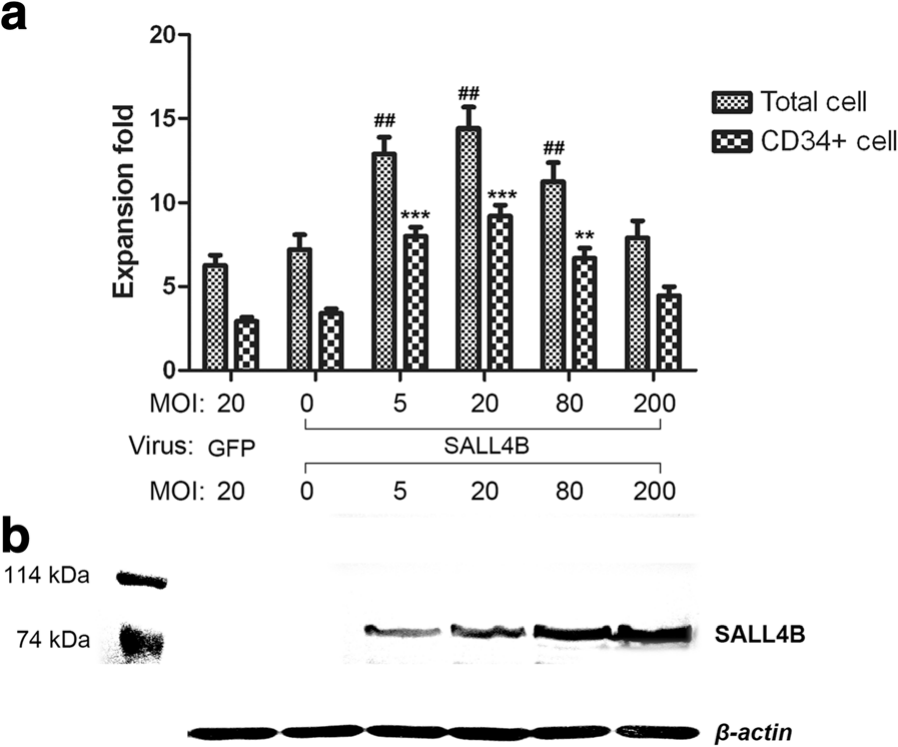
Determination of optimal MOI for GFP-Sall4B lentivirus in primate CD34^+^ cells. Nonhuman primate BM CD34^+^ cells transduced with GFP-control and GFP-Sall4B lentivirus at MOI of 0, 5, 20, 80, or 200 for 12 hours, and cultured for 9 days. **a** On day 9, total cell number was counted and CD34^+^ proportions were obtained by flow cytometry. The fold expansion of total nucleated cells and CD34^+^ cells was calculated based on the cell number on both day 0 and day 9. Data presented as mean ± SD. ^##^ *P* < 0.01, vs Sall4B at MOI = 0 group (total cell); **P < 0.01, ***P < 0.001, vs Sall4B at MOI = 0 group (CD34^+^ cell). One-way ANOVA followed by Dunnett’s multiple comparison test. **b** Sall4B polyclonal antibody was used to detect Sall4B protein in cell lysates from all groups on day 9. *β-actin* was used as the loading control. Sall4B protein bands were detected at 76 kDa in a dose-dependent manner, with MOI at 5, 20, 80, or 200. *GFP* green fluorescent protein, *MOI* multiplicity of infection

The determination of optimal MOI was required due to the toxicity of lentivirus and unknown side effects of SALL4B protein overexpression in cells. Safe doses were determined for GFP-Sall4B lentivirus group with MOI at 0, 5, 20, 80, or 200, and for GFP-control lentivirus group with MOI at 20. The total nucleated cells and CD34^+^ cells in each group were observed on day 9, as shown in Fig. 4a. As the MOI values of the GFP-Sall4B lentivirus group increased from 0 to 20, the total cell expansion fold increased. However, the fold expansion of total cells and CD34^+^ cells decreased rapidly when the MOI value was above 20. Western blot of Sall4B protein expression was performed to observe whether Sall4B protein expression levels were accompanied by variations in MOI, as shown in Fig. 4b. An increase of Sall4B protein in total cell lysate was observed, indicating that the GFP-Sall4B lentivirus efficiently transduced primate CD34^+^ cells.

### Characterization of Sall4B-transduced CD34^+^ cells ex vivo

**Fig. 5:**
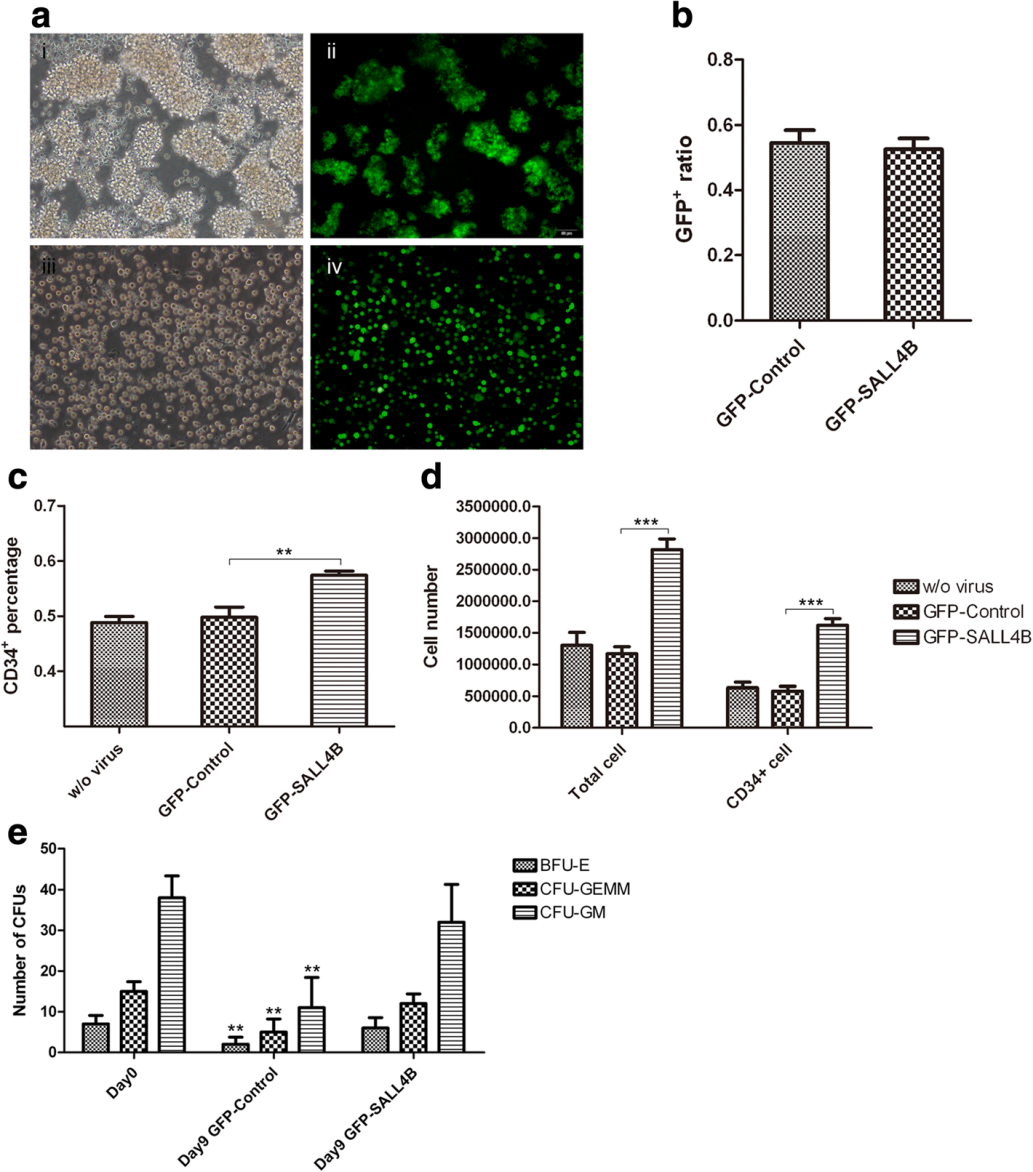
Expansion and characterization of Sall4B-transduced primate CD34^+^ cells. **a** Bright-field and fluorescent images of nonhuman primate BM CD34^+^ cells transduced with GFP-Sall4B lentivirus (*i* and *ii*, 20 ×) or GFP-control lentivirus (*iii* and *iv*, 20 ×) on day 9. The GFP-control CD34^+^ primate cell clusters were distinguishable from GFP-Sall4B lentivirus groups. **b** Flow cytometry measured the proportion of GFP-positive cells transduced with GFP-Sall4B or GFP-control lentivirus. **c** CD34^+^ percentage measured by flow cytometry on day 9 after transduction. **P < 0.01, GFP-Sall4B group vs GFP-control group. **d** On day 9, total nucleated cells were counted and CD34^+^ cells were calculated from the total cell number and measured CD34^+^ percentage. ***P < 0.001, GFP-Sall4B group vs GFP-control group. **e** CFU colonies formed from CD34^+^ cells transduced with GFP-Sall4B lentivirus or GFP-control lentivirus on day 9. Freshly purified CD34^+^ cells from day 0 were used as the positive control. The CFU colonies were counted on day 16 after cells were cultured in CFU MethoCult Media. **P < 0.01, vs freshly purified CD34^+^ cells from day 0. One-way ANOVA followed by Dunnett’s multiple comparison test. *GFP* green fluorescent protein, *w/o* without, *BFU-E* erythroid burst-forming unit, *CFU-GEMM* granulocyte–erythrocyte–macrophage–megakaryocyte colony-forming unit; *CFU-GM* granulocyte–monocyte colony-forming unit

Primate CD34^+^ cells transduced with GFP-Sall4B at MOI of 20, or with GFP-control lentivirus and saline groups as controls, were cultured for 9 days. Typical GFP-CD34^+^ cell clusters were observed (Fig. 5a, i and ii). In contrast, cells were not formed in a large cluster for control groups because only a small cluster or single GFP cell was visible (Fig. 5a, iii and iv). The efficiency of lentivirus transduction was 55 ± 4.87 % (Fig. 5b). On day 9, fold expansion was 14.4 ± 1.80 (total nucleated cells) and 9.21 ± 1.94 (CD34^+^ cells) for the Sall4B-transduced group, and was 6.26 ± 0.87 (total nucleated cells) and 2.95 ± 0.77 (CD34^+^ cells) for the GFP-control group (Fig. 5d). In addition, the CD34^+^ cell ratio in the Sall4B group was significantly higher than in the other two control groups (Fig. 5c). These results suggested that Sall4B lentivirus was capable of maintaining the nonhuman primate HSC properties, as well as enhancing cell proliferation. CFU assay examined the repopulation and multilineage differentiation of expanded CD34^+^ cells transduced by Sall4B lentivirus on day 9. The Sall4B-transduced cells, as well as freshly isolated primate CD34^+^ cells from day 0, formed various colonies of CFU-GM, CFU-GEMM, and BFU-E. However, significantly less colonies were formed for the GFP-control group (Fig. 5e). The results indicated that Sall4B lentivirus maintained stemness for expanded nonhuman primate CD34^+^ cells.

### In-vivo transplantation and repopulation assay in NOD/SCID mice

**Fig. 6:**
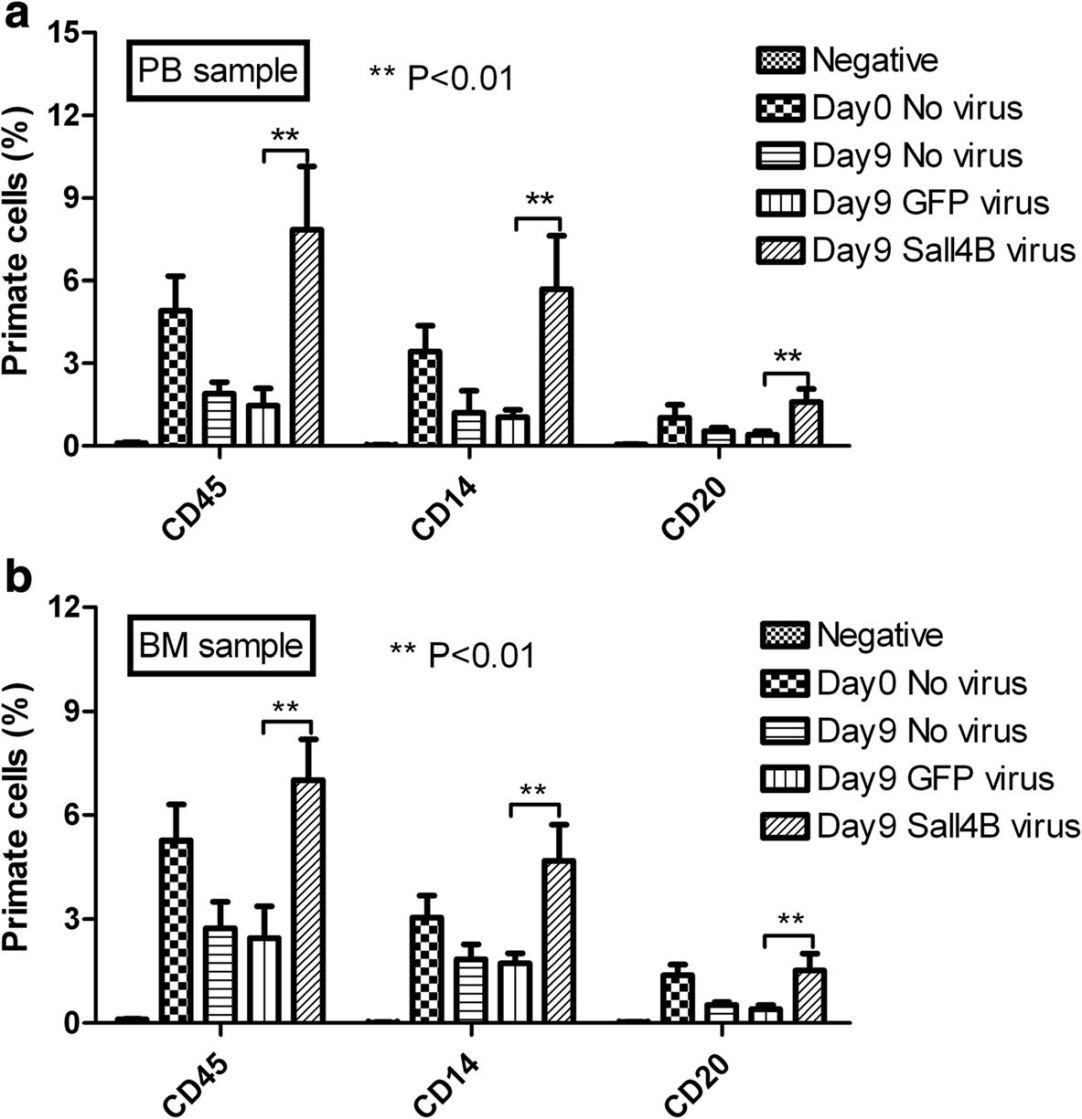
Nonhuman primate cells in PB and BM of NOD/SCID mice transplanted with various stages of CD34^+^ cells. Eight weeks after intravenous primate CD34^+^ transplantation in mice, primate CD45^+^, CD14^+^, and CD20^+^ cells were analyzed in the PB (**a**) and BM (**b**) of mice transplanted with unexpanded primate CD34^+^ cells, 9-day expanded CD34^+^ cells, 9-day GFP-control transduced CD34^+^ cells, and 9-day Sall4B-GFP transduced CD34^+^ cells, respectively. Normal saline was injected as the vehicle control. Data represent mean ± SD, *n* = 16. **P < 0.01. One-way ANOVA followed by Dunnett’s multiple comparison test. *BM* bone marrow, *GFP* green fluorescent protein, *PB* peripheral blood

The engraftment of nonhuman primate hematopoietic cells was analyzed in five groups of irradiated NOD/SCID mice. As shown in Fig. 6, differentiated primate cell clusters were not detected by flow cytometry in the PB and BM cells of negative control mice (saline) in week 8 post transplantation. On the other hand, the primate CD45 marker was detected at 4.92 ± 1.24 %, 1.89 ± 0.43 %, 1.47 ± 0.62 %, and 7.84 ± 2.31 % in the PB of groups transplanted with day 0 primate CD34^+^ cells, day 9 expanded primate CD34^+^ cells, day 9 GFP-control lentiviral transduced CD34^+^ cells, and day 9 GFP-Sall4B lentiviral transduced CD34^+^ cells, respectively (Fig. 6a). Primate myeloid lineage marker CD14 and lymphoid lineage marker CD20 could also be detected in the cell transplanted groups (Fig. 6a). In mouse BM, primate CD45 marker was exhibited at 5.27 ± 1.04 %, 2.73 ± 0.77 %, 2.45 ± 0.92 %, and 7.01 ± 1.18 % in day 0 primate CD34^+^ cells, day 9 expanded primate CD34^+^ cells, day 9 GFP-control lentiviral transduced CD34^+^ cells, and day 9 GFP-Sall4B lentiviral transduced CD34^+^ cells, respectively (Fig. 6b). Primate lineage markers CD14 and CD20 were also detected at different levels in four cell transplanted groups (Fig. 6b). These results indicated that GFP-Sall4B transduced cells had the ability to control their differentiation, repopulation, and peripheral cell output, when compared with GFP-control transduced cells or normal expanded CD34^+^ cells on day 9 in vivo*.* The cell population expanded by Sall4B ex vivo for 9 days was revealed to have priority in repopulating primate cells in NOD/SCID mice. Moreover, the SRC frequency was found to be significantly enhanced by stem cell factor Sall4B through limiting dilution analysis, which demonstrated a 2.27-fold increased SRC frequency in mice that received 9-day-old SALL4B expressed primate CD34^+^ cells (59 per 10^6^ starting CD34^+^ cells) compared with those receiving 9-day-old lentivirus control primate CD34^+^ cells (26 per 10^6^ starting CD34^+^ cells), and a 1.59-fold increased compared with those unexpanded primate CD34^+^ cells (37 per 10^6^ CD34^+^ starting cells) at 8 weeks post transplantation. These results suggested that the Sall4B could be effective in expanding nonhuman primate CD34^+^ cells.

**Fig. 7:**
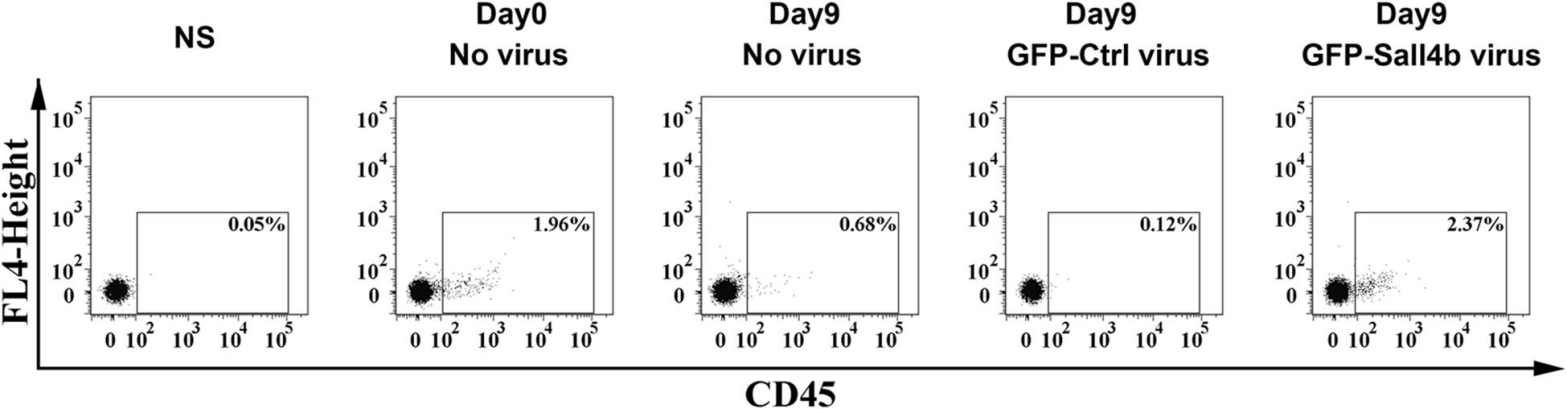
Secondary BM transplantation in NOD/SCID mice. Representative flow cytometric analysis of the mouse BM CD45^+^ cell population from five groups in week 8 post secondary transplantation. *NS* normal saline, *GFP* green fluorescent protein

To further determine whether Sall4B-transduced cells bear long-term engraftment, secondary BM transplantations were performed. Flow cytometry analysis of mouse BM cells harvested from femurs in week 8 post secondary transplantation showed that primate CD45^+^ cells could be detected at 1.87 ± 0.73 %, 0.53 ± 0.22 %, 0.21 ± 0.12 %, and 2.05 ± 1.03 % in groups transplanted with day 0 primate CD34^+^ cells, day 9 expanded primate CD34^+^ cells, day 9 GFP-control lentiviral transduced CD34^+^ cells, and day 9 GFP-Sall4B lentiviral transduced CD34^+^ cells, respectively (Fig. 7). The result demonstrated that Sall4B-transduced CD34^+^ cells could be successfully transplanted from one animal BM to another on day 9. It was verified that Sall4B-transduced CD34^+^ cells possessed long-term engraftable property.

## Discussion

Multiple protein factors, including transcription factors, epigenetic modifiers, and cell cycle regulators, play an important role in the regulation of HSC self-renewal [25, 26]. Our previous studies demonstrated that transcription factor Sall4B was a robust stimulator for human and mouse HSC expansion when combined with cytokines (SCF, TPO, and Flt-3 L) [1]. TAT-Sall4B protein was able to enhance the short-term and long-term engraftment of human umbilical cord blood CD34^+^ cells in NOD/SCID mice [27]. Although the genes between human and nonhuman primate Sall4B are 97 % homologous, it is still unknown whether human Sall4B possesses the same expansion capacity as nonhuman primate CD34^+^ cells. In the current study, we have conducted ex-vivo expansion of CD34^+^ cells from primate BM and xenogenic transplantation in the NOD/SCID mouse model.

Our previous data also indicated that Sall4B enhanced stem cell growth only in conjunction with the cytokines (SCF and TPO) in its environment [1]. In the current study, SCF, TPO, and Flt-3 L were employed as basic cytokine cocktails along with lentiviral Sall4B to support cell growth. We found that CD34^+^ cells were in a resting state when lentiviral Sall4B was transduced without any additional cytokines during the first 2-day or 3-day culture, which led to cell death. However, there were some living cells observed on day 9. On the other hand, with the addition of three cytokines, 3.14 ± 0.57-fold CD34^+^ cell expansion, along with 2.95 ± 0.77-fold expansion for the GFP-control lentivirus group, and 9.21 ± 1.94-fold expansion for the GFP-Sall4B lentivirus group were obtained. This result indicated that GFP-control lentivirus transduction would have no effect on cell proliferation, and that lentiviral cells with Sall4B significantly enhanced primate CD34^+^ cell expansion as compared with the other two control groups. The percentage of CD34^+^ cells in the GFP-Sall4B group was 9.12 ± 5.64 % higher than either the GFP-control group or the cytokine only group; thus implicating that Sall4B not only increased CD34^+^ cell proliferation, but also maintained the stemness of HSCs. This result was also confirmed by CFU-GEMM assay, which suggested that day 9 culture cells still possessed multilineage differentiation ability due to Sall4B overexpression, as in freshly purified CD34^+^ cells.

We also found that an appropriately balanced level of Sall4B expression was essential in maintaining HSC self-renewal, multipotency, and differentiation. There were two key factors that might influence Sall4B protein expression levels in primate cells: lentivirus MOI and transduction time. By comparing the total cell and CD34^+^ cell fold proliferation between each MOI group, GFP-Sall4B lentivirus was added at different MOIs to determine optimal MOIs,. The primate cell expansion was improved as MOI increased from 0 to 20; however, cells could lose expansion capacity when MOI continually increased from 20 to 200. This result was consistent with our previously proposed study model [23]. The different lentivirus transduction time points were also compared at this time. We optimized for high transduction efficiency and low lentiviral cell toxicity; as a result, 12 hours was selected as the optimal transduction time. Sall4B protein could be detected in a dose-dependent fashion by western blot analysis, which indicated that Sall4B expression levels could be controlled by optimizing the MOI and transduction time.

Certain novel transcription factors showed the capability to expand HSCs, such as HoxB4. However, lentivirus transduction of these genes could cause expanded cells to lose long-term repopulation potential [28]. Our secondary xenotransplantation results in mice showed that Sall4B-transduced nonhuman primate CD34^+^ cells on day 9 retained both short-term and long-term engraftable property, as in freshly isolated CD34^+^ cells. Furthermore, there was no evidence of leukemia in transplanted mice more than 6 months after transplantation, indicating that the expanded Sall4B-transduced CD34^+^ cells were safe in vivo.

We tried to purify TAT-Sall4B protein in bacteria [1] and Sf9 insect cell expression systems [16, 29]. The protein yield was too low for studies in mice. GFP-Sall4B lentivirus functioned as both transduced vector and marker for verifying Sall4B efficacy on primate CD34^+^ cells in vivo. More efforts should be made to improve the yield of TAT-Sall4B protein production on a large scale.

The published human data were based on murine models. As we all know, the overwhelming homology between humans and primates make the primate model a valuable one for preclinical studies, including HSC transplantation. However, a transplant human cell into nonhuman primate recipes is infeasible due to the inevitable immunological rejection. Thus, autotransplantation is an alternative to evaluate the expanded HSC in nonhuman primate model. This is the first study to show that CD34^+^ cells derived from nonhuman primate could be efficiently expanded by overexpressing Sall4B, which would serve as a footstone for further preclinical studies in nonhuman primate autologous transplantation and clinical applications.

## Conclusions

Nonhuman primate CD34^+^ cell expansion capability could be effectively enhanced by Sall4B lentiviral overexpression, which provides a new perspective on mechanisms of rapid stem cell proliferation. Additionally, it would provide preclinical information that would be helpful towards autologous transplantation in the clinic.

## Abbreviations

3Rs: Regulations of replacement, refinement, and reduction
AML: Acute myeloid leukemia
APC: Allophycocyanin
BFU-E: Erythroid burst-forming unit
BM: Bone marrow
CFU: Colony-forming unit
CFU-G: Granulocyte colony-forming unit
CFU-GEMM: Granulocyte–erythrocyte–macrophage–megakaryocyte colony-forming unit
CFU-GM: Granulocyte–monocyte colony-forming unit
CFU-M: Megakaryocyte colony-forming unit
CO_2_: Carbon dioxide
FITC: Fluorescein isothiocyanate
Flt-3L: Fms-related tyrosine kinase 3 ligand
FSC: Forward scatter
GFP: Green fluorescent protein
HoxB4: Homeobox B4
HSC: Hematopoietic stem cell
HSPC: Hematopoietic stem/progenitor cell
MACS: Magnetic-activated cell sorting
MNC: Mononuclear cell
MOI: Multiplicity of infection
NOD/SCID: Nonobese diabetic/severe combined immunodeficiency
PB: Peripheral blood
PBS: Phosphate-buffered saline
PE: Phycoerythrin
RT: Room temperature
SCF: Stem cell factor
SRC: SCID repopulating cells
SSC: Side scatter
TPO: Thrombopoietin
